# Wavelet Coherence Analysis of Post-Stroke Intermuscular Coupling Modulated by Myoelectric-Controlled Interfaces

**DOI:** 10.3390/bioengineering11080802

**Published:** 2024-08-08

**Authors:** Xinyi He, Wenbo Sun, Rong Song, Weiling Xu

**Affiliations:** 1 School of Biomedical Engineering, Shenzhen Campus of Sun Yat-sen University, Shenzhen 518107, China; hexy53@mail2.sysu.edu.cn (X.H.); sunwb3@mail2.sysu.edu.cn (W.S.); 2 Shenzhen Research Institute of Sun Yat-sen University, Shenzhen 518107, China; 3 School of Information Science and Technology, Zhongkai University of Agriculture and Engineering, Guangzhou 510408, China

**Keywords:** stroke, intermuscular coupling, dimensionality, feedback, wavelet coherence, myoelectric-controlled interface

## Abstract

Intermuscular coupling reflects the corticospinal interaction associated with the control of muscles. Nevertheless, the deterioration of intermuscular coupling caused by stroke has not received much attention. The purpose of this study was to investigate the effect of myoelectric-controlled interface (MCI) dimensionality on the intermuscular coupling after stroke. In total, ten age-matched controls and eight stroke patients were recruited and executed elbow tracking tasks within 1D or 2D MCI. Movement performance was quantified using the root mean square error (RMSE). Wavelet coherence was used to analyze the intermuscular coupling in alpha band (8–12 Hz) and beta band (15–35 Hz). The results found that smaller RMSE of antagonist muscles was observed in both groups within 2D MCI compared to 1D MCI. The alpha-band wavelet coherence was significantly lower in the patients compared to the controls during elbow extension. Furthermore, a decreased alpha-band and beta-band wavelet coherence was observed in the controls and stroke patients, as the dimensionality of MCI increased. These results may suggest that stroke-related neural impairments deteriorate the motor performance and intermuscular coordination pattern, and, further, that MCI holds promise as a novel effective tool for rehabilitation through the direct modulation of muscle activation pattern.

## 1. Introduction

A coordinated movement was inseparable from the complex interactions between behavioral goals, neural control, and musculoskeletal apparatus [1]. This interaction was exemplified by a tightly intertwined functional relationship between the agonist and antagonist muscles. However, loss of neurological function after stroke, such as the common injury of the corticospinal tract [2], could impede a patient’s ability to initiate movement, thereby leading to a compromise in the movement performance. In general, the restricted motor ability in patients was associated with the impaired muscle coordination patterns [3,4,5]. Previous studies have observed the reduced intermuscular coupling in stroke versus age-matched controls [6,7,8,9], which was usually contributed to the motor deficits. It is likely to modulate the intermuscular coupling to improve the motor function [10,11,12]. The factors linked to the poor coordination after stroke included abnormal muscle activation patterns [13], disruption in the recruitment of agonist muscles and the inhibition of antagonist muscles [14], weakened corticomuscular coupling [15], and so on. Despite the observed diminished functional coupling between cortex and muscles in patients, the changes in the intermuscular coupling due to neurological impairments after stroke have not been fully elucidated yet.

An effective method to quantify the intermuscular coupling could be to investigate the oscillatory activity between the corresponding muscles from the electromyogram (EMG) signal. Conventional coherence analysis provided the estimates of the synchronous oscillations between the EMG and EMG signals in the frequency domain [16]. Due to its reliance on the time-stable signals, however, coherence analysis fails to capture the time-varying characteristics between the nonstationary EMG signals of dynamic process. By contrast, wavelet coherence [17], utilizing wavelet transform to provide an accurate time–frequency representations of signals, allows for the efficient identification of short-term coupling between signals, especially for the nonstationary electrophysiological signals. A previous study used wavelet coherence on the electroencephalogram (EEG) signals to examine the neural coactivation among brain regions [18]. Furthermore, the wavelet coherence in the different frequency segments reflected the various neurophysiological information [8]. Rhythmic activity could be recorded from the motor cortex to muscles in the human subjects in the alpha (8–12 Hz) [19] and beta (15–35 Hz) bands [20,21]. The alpha-band intermuscular coherence was considered to be associated with the control mechanism of the slow movements [22], and the beta-band intermuscular coherence was thought to reflect a contribution from the corticospinal drive [23,24]. Therefore, wavelet coherence served as a good option for the measurement of dynamic neural interactions. This method was introduced for a more comprehensive assessment of intermuscular coupling between the EMG signals, offering insights into the neurophysiological information represented in different frequency segments.

Addressing the abnormal muscle coordination patterns was a potential new avenue for stroke rehabilitation. Generally, stroke altered muscle coordination and exaggeratedly activated antagonist [25], thereby led to the impaired motor function. The conventional physical therapy could contribute to the part recovery of their motor function but still with a residual disability in performing the fine movement [26,27]. As previous studies showed, intermuscular coupling was considered to play a crucial part in the fine motor control and the maintenance of the neuromuscular performance [28,29,30,31]. Consequently, the modulation of intermuscular coupling may contribute to executing the fine movement and further facilitating the motor recovery. Previous studies have indicated that visual feedback exhibited influence on the intermuscular coupling. Chen et al. [32] recently noted that subjects showed a reduction in beta-band motor unit coherence of the first dorsal interosseus with the enhancement of visual feedback. Also, Nguyen et al. [33] illustrated a reduction in the coherence between biceps muscles during bilateral elbow extension with supplemental visual information. Subsequently, a myoelectric-controlled interface (MCI), a kind of visual feedback tool conveying information of muscle activation, was introduced to modulate the abnormal muscle activation with the control of EMG signals [10,34]. Stroke patients have constructed to utilize the MCI to facilitate relearning and readapting the normal muscle coordination pattern [35,36]. For example, Jian et al. developed a novel MCI training paradigm specifically for the stroke patients, successfully mitigating the abnormal antagonist activation due to the impaired inhibition mechanisms [37,38]. The dimensionality of MCI, containing different visual feedback, was an important factor that could affect movement performance. Previous studies have found that the better performance was usually related with the higher dimensionality of visual feedback [39,40]. Despite previous reports confirming the ability of MCI to modulate muscle activation, the effect of MCI dimensionality on the coupling between agonist and antagonist muscles has not been fully elucidated.

This study sought to investigate the effect of MCI dimensionality on the intermuscular coupling in the control subjects and stroke patients during a tracking task. Changes in the dimensionality within the myoelectric-controlled interface (MCI) were associated with varying visual feedback regarding muscle activation. Due to its superior capability in detecting the coupling between signals during dynamic processes, wavelet coherence was employed to quantify intermuscular coupling between the biceps and triceps. It was hypothesized that stroke patients, compared to the controls, exhibited distinct intermuscular coupling patterns between agonist and antagonist muscles. It was also predicted that the increased dimensionality of MCI would lead to the intermuscular decoupling, suggesting that MCI training could be designed as a novel therapy to favor the functional recovery of muscle coupling in post-stroke patients.

## 2. Methods

### 2.1. Participants

This study recruited stroke patients with the following inclusion criteria: (1) a minimum of one-month onset; (2) normal hearing and normal or corrected-to-normal vision; (3) absence of significant muscle tone increase (modified Ashworth scale, MAS > 2); (4) satisfactory performance on cognitive functions (mini-mental state examination, MMSE > 23). In total, eight stroke patients (two females and six males; mean age: 47.5 ± 16.2 years) and ten right-handed age-matched controls (five females and five males; mean age: 53 ± 6.3 years) joined in the study. Table 1 presents detailed information about the stroke patients. Prior to the study, all participants were asked to provide written informed consent. The study was approved by the Ethics Committee of the Guangdong Work Injury Rehabilitation Center.

### 2.2. Experimental Procedure

Before the experiment, participants were asked to sit comfortably in front of a computer screen for a short round of practice. Healthy participants were directed to position their dominant arm on the horizontal armrest with the elbow flexed at 90° and the shoulder abducted at 90° [41]. Stroke patients were instructed to place their impaired arm in the same configuration. All participants were required to grasp a handle attached to the armrest, adjustable to their forearm length. Belts were utilized to secure the elbow, forearm, and wrist in place.

Two circular silver–silver chloride (Ag–AgCl) electrodes were affixed in parallel to the biceps and triceps bellies after skin preparation with alcohol. Surface electromyogram (EMG) signals were recorded using a customized EMG amplifier, sampled by a data converter (DAQ USB-6341, National Instrument Corporation, Austin, TX, USA) with 16-bit resolution at a rate of 1000 Hz, and stored in a computer through a LabVIEW program (LabVIEW 2012, National Instruments Corporation, Austin, TX, USA). The screen in front of the participants displayed the myoelectric-controlled interface (MCI), containing a two-dimensional coordinate system and two square cursors (1 cm × 1 cm in size): a red cursor for the target and a green cursor for user control. The normalization activation of the triceps and biceps was mapped to the *x*-axis and *y*-axis of the two-dimensional coordinate system, respectively.

**Figure 1:**
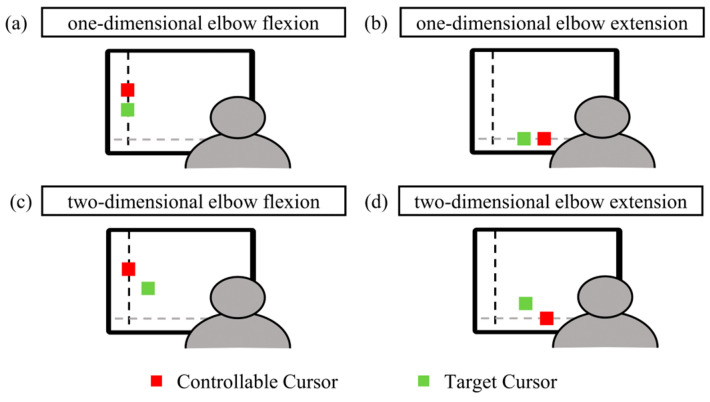
Example scenes of four types of visual feedback. One-dimensional MCI, in which the red cursor represents the target cursor, and the green cursor represents the manipulable cursor moving along the dashed line during flexion (**a**) and extension (**b**); two-dimensional MCI, in which the green cursor moves in the 2D coordinate system during flexion (**c**) and extension (**d**).

Prior to the experiment, participants engaged in a short practice session, refining their ability to control a green cursor to closely follow a red target. This movement of the green cursor was achieved through the activation of the biceps and triceps. At the experimental beginning, participants executed three 5 s maximal voluntary contractions (MVCs) tasks, incorporating a 2 min interval between each trial. The maximal EMG amplitude over three trials was recorded and utilized to normalize the activation of the biceps and triceps for subsequent tracking tasks. To mitigate fatigue, the maximal muscle activation was set at 15% MVC [42]. Subsequently, participants were directed to execute isometric elbow flexion and extension to track the target cursor under varying visual feedback conditions (one-dimensional and two-dimensional, denoted as 1D and 2D, respectively). With one-dimensional visual feedback of agonist activation, the manipulable cursor was restricted to movement along either the *x*-axis or *y*-axis, reflecting the control solely by the activation of agonist muscles. With two-dimensional visual feedback of agonist and antagonist activation, the manipulable cursor moved across the 2D coordinate plane, reflecting the control concurrently by the activation of both agonist and antagonist muscles. Figure 1a,c depicts the elbow flexion task, illustrating the triceps as the agonist muscles and the biceps as the antagonist muscles. In Figure 1b,d, the elbow extension task is presented, with biceps serving as the agonist muscles and triceps as the antagonist muscles.

In the tracking task, the target cursor executed a linear movement from the origin to the end of the *x*-axis (15% MVC, 0) or from the origin to the end of the *y*-axis (0, 15% MVC) continuously at a constant speed. Participants were instructed to incrementally activate the agonist muscles from 0 to 15% MVC within 0–5 s, followed by a gradual relaxation and return to the origin within the next 5 s. Each trial spanned 30 s and encompassed three repetitions of the aforementioned process. Participants were randomized to perform a total of 20 trials, with a 30 s break interposed between each trial.

### 2.3. Wavelet Coherence

To examine the coupling between nonstationary EMG signals, the wavelet coherence [43] was introduced, which is a measurement of the coherency between two time series in the time–frequency space, defined as follows:

(1) $$WC_{xy}^{2}\left(t,f\right)=\frac{{\left|{SW}_{xy}(t,f)\right|}^{2}}{{SW}_{xx}\left(t,f\right)\times {SW}_{yy}(t,f)}$$

where ${SW}_{xy}\left(t,f\right)$ is the cross-wavelet spectrum between $x\left(t\right)$ and y(t), and ${SW}_{xx}\left(t,f\right)\times {SW}_{yy}(t,f)$ are the auto-wavelet spectrum of $x\left(t\right)$ and y(t).

Then, the binary wavelet coherence ${BWC}_{xy}(t,f)$ in the time–frequency domain was calculated as follows:

(2) $${BWC}_{xy}\left(t,f\right)=\left\{\begin{array}{ll} 1 & {WC}_{xy}(t,f)-TS>0\\ 0 & {WC}_{xy}\left(t,f\right)-TS\leq 0 \end{array}\right.$$

where the threshold, denoted as TS, serves as a criterion for binary wavelet coherence, facilitating the identification of synchronous oscillations. A positive outcome, evident when the wavelet coherence value exceeds TS, signifies the statistical significance (*p* = 0.05) of synchronous oscillations within the examined time series. Conversely, a negative outcome indicates the absence of synchronous oscillations between the time series. For the purposes of this investigation, the predetermined threshold TS was precisely specified at 0.6039 [8].

The wavelet coherence values were divided into distinct frequency segments (alpha band: 8–12 Hz, beta band: 15–35 Hz [20]) for a more detailed analysis of the functional coupling between biceps and triceps. Based on the average of binary wavelet coherence ${BWC}_{xy}(t,f)$ within the frequency domain, the averaging wavelet coherence in designated frequency segments ${AFWC}_{xy}(t)$ was calculated through the subsequent equation:

(3) $${AFWC}_{xy}\left(t\right)=\frac{1}{h-g}\int_{g}^{h}{BWC}_{xy}\left(t,f\right)df$$

where *g* delineates the lower bounds and *h* delineates the upper bounds of the frequency segments.

### 2.4. Statistical Analysis

EMG signals were firstly high-pass filtered at 5 Hz using a fourth-order Butterworth band-pass filter, followed by a notch filter at 50 Hz to eliminate low-frequency artifacts and power-line interference. The averaging binary wavelet coherence ${AFWC}_{xy}$ in various frequency segments of EMG signals was analyzed in MATLAB (MathWorks™ Inc., Natick, MA, USA, https://www.mathworks.com/products/matlab.html (accessed on 5 August 2024)).

The root mean square error (RMSE) between the target cursor and tracking cursor was employed to assess the overall motor performance [44]. According to the two-dimensional coordinate system, RMSE in the *x*-axis and *y*-axis was calculated, respectively. The smaller RMSE was linked with the better tracking performance. The RMSE of each trial was calculated and was averaged as the behavioral metric for further analysis.

Statistical analyses were conducted using SPSS 26.0 (SPSS Inc., Chicago, IL, USA). To assess the effect of the group (controls and stroke patients) and visual dimensionality, a Mann–Whitney U-test was employed on RMSE values and alpha-band and beta-band wavelet coherence values. Also, a Wilcoxon signed rank test was used to analyze the effect of visual dimensionality (1D and 2D) within the same group. Statistically, the significant level was set at *p* < 0.05.

## 3. Results

**Figure 2:**
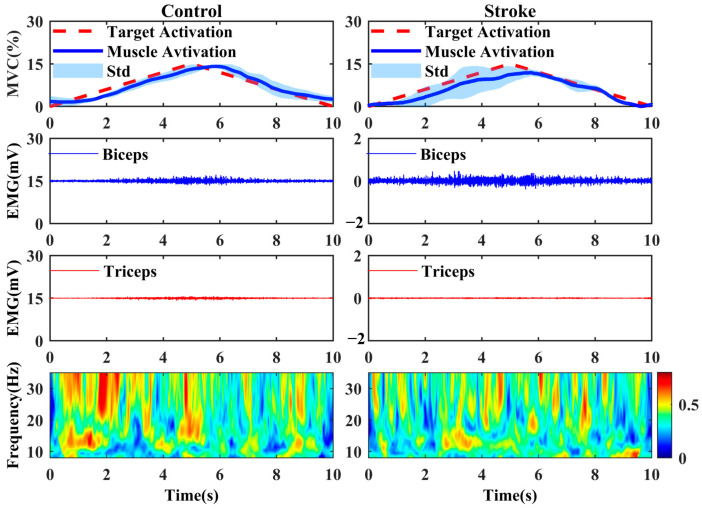
Typical muscle activation, EMG signals of the biceps and triceps, and the binary wavelet coherence in the alpha and beta bands recorded during an 1D elbow flexion task. Control: a healthy subject; stroke: a stroke patient. Target activation: 15% MVC; Std: the standard deviation calculated from target activation and muscle activation.

Figure 2 displays the distinctive muscle activation situations of agonist muscles, accompanied by the electromyographic (EMG) signals of the biceps and triceps. Moreover, it illustrates the binary wavelet coherence in the alpha and beta band during an elbow flexion task within the one-dimensional interface (1D-MCI). The presented data were obtained from two representative in the controls and stroke patients.

**Figure 3:**
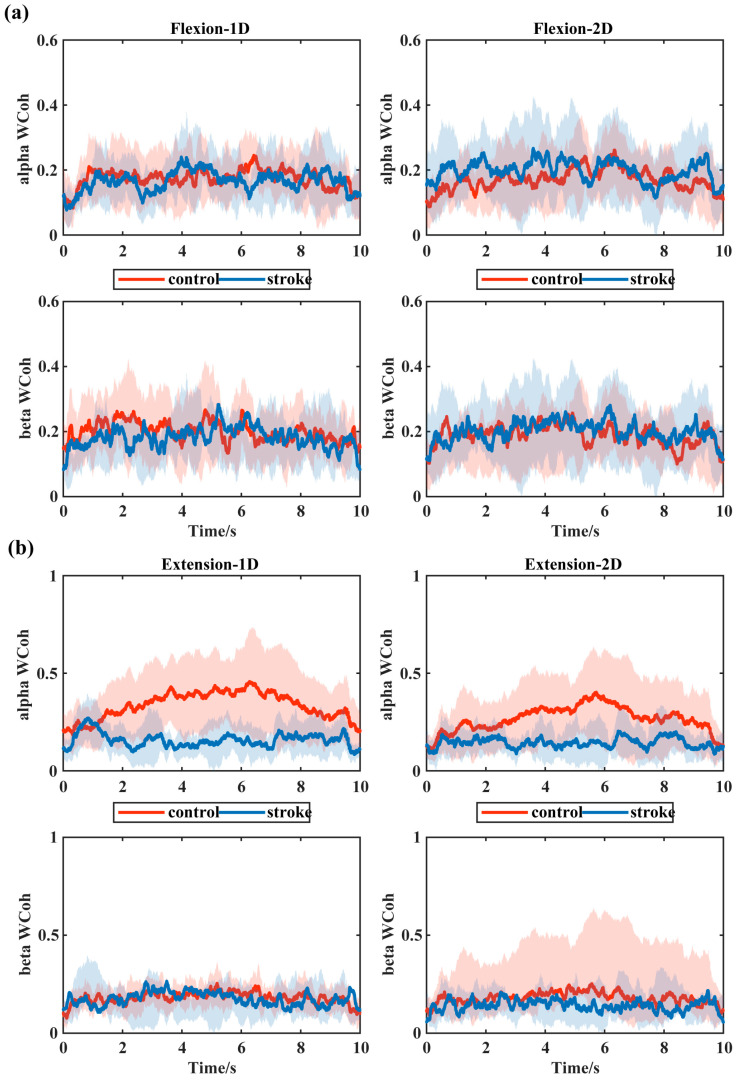
Averaging binary wavelet coherence AFWC between biceps and triceps in the alpha band and beta band between the controls (the red line) and the patients (the blue line). (**a**) During the elbow flexion task within both 1D and 2D MCI; (**b**) during the elbow extension task within both 1D and 2D MCI.

Figure 3 displays the averaging binary wavelet coherence ${AFWC}_{xy}$ in the alpha and beta bands between biceps and triceps in the elbow flexion and extension task within both 1D and 2D MCI. The wavelet coherence in the alpha and beta rhythm exhibited similar temporal changes between controls and stroke patients in the elbow flexion task. However, in the extension task, the wavelet coherence demonstrated different temporal changes. The controls exhibited higher ${AFWC}_{xy}$ in the alpha band than stroke patients within both 1D and 2D MCI. However, in the beta band, both the controls and stroke patients exhibited similar lower ${AFWC}_{xy}$ in both 1D and 2D MCI conditions. These results indicate that regardless of MCI dimensionality, controls and stroke patients adopted a similar functional coupling pattern to execute the elbow flexion task. However, during elbow extension, stroke patients consistently exhibited a lower functional coupling between biceps and triceps compared to the controls.

**Figure 4:**
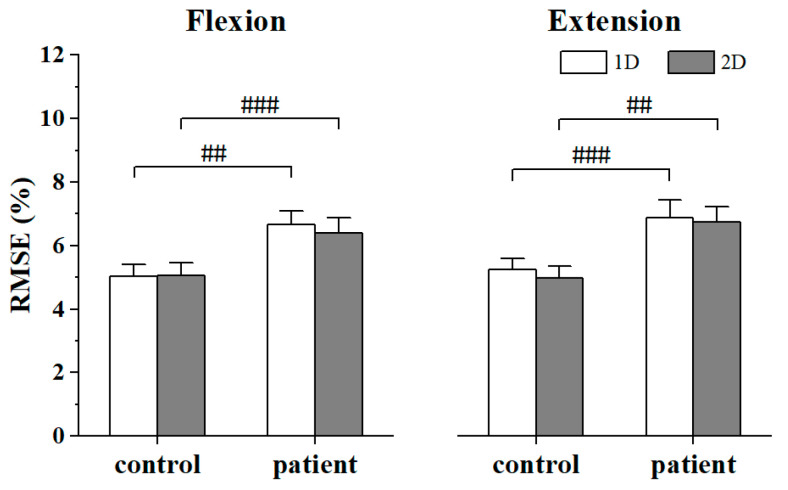
Bar plots of mean RMSE in each group within 1D and 2D MCI during the elbow flexion and extension. The number sign (#) indicates significant difference between controls and stroke patients. The error bar shows standard error.

**Figure 5:**
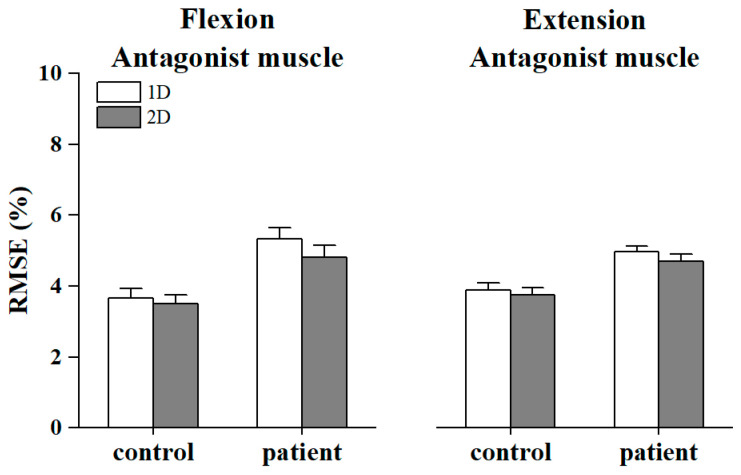
Bar plots of mean RMSE of antagonist muscles mapped to *x*-axis or *y*-axis, respectively, in each condition during the elbow flexion (**a**) and extension (**b**). The error bar shows standard error.

Figure 4 displays the bar plots of RMSE of agonist and antagonist muscles in two groups under both conditions, and Figure 5 displays the bar plots of RMSE of antagonist muscles along the *x*-axis and *y*-axis, respectively, in both groups. The Mann–Whitney U-test revealed that the RMSE values of the controls were significantly smaller than those of patients during all tasks (Flexion_1D: *p* = 0.043; Flexion_2D: *p* = 0.004; Extension_1D: *p* = 0.001; Extension_2D: *p* = 0.003, Figure 4). Regarding the RMSE of antagonist muscles along the *x*-axis and *y*-axis, the Wilcoxon signed rank test showed that compared to 1D MCI, RMSE decreased in the patients within 2D MCI during elbow flexion. In addition, there was no significant results between other RMSE, though it was observed that compared to 1D MCI, RMSE consistently decreased within 2D MCI.

**Figure 6:**
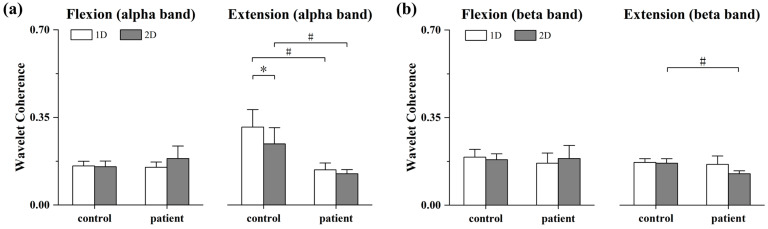
Bar plots of mean alpha-band and beta-band binary wavelet coherence in both groups within 1D and 2D MCI during the elbow flexion (**a**) and extension (**b**). The asterisk (*) indicates significant pairwise difference between 1D and 2D MCI, and the number sign (#) indicates significant difference between controls and stroke patients. The error bar shows standard error.

For the alpha-band coherence, there was no significant differences between the controls and patients during the elbow flexion task, whereas a significantly higher coherence in the controls than the patients was observed within both 1D and 2D MCI (1D: *p* = 0.004, 2D: *p* = 0.034, Figure 6a). Also, there was a significant reduction in alpha-band coherence of the controls as the dimensionality increased in the elbow extension task (*p* = 0.005, Figure 6a). In addition, for the beta-band coherence, the controls showed a significantly higher coherence than the patients in the 2D MCI during elbow extension. Figure 6 displays the mean coherence values in the same group within different dimensional MCIs.

## 4. Discussion

In this study, wavelet coherence was utilized to analyze the intermuscular coupling between agonist and antagonist muscles within nonoverlapping frequency segments within both 1D and 2D MCI. The differences in tracking errors and intermuscular coherence between controls and stroke patients were systematically assessed to investigate how stroke affected the motor performance as well as intermuscular coordination.

### 4.1. Effect of Stroke on the Intermuscular Coupling of the Synergistic Muscles

Compared with healthy controls, stroke patients showed greater tracking errors in each condition. Our findings provided additional evidence suggesting that patients exhibited a poor control of muscle activation [13,45]. A coordinated movement consistently exhibited characteristics that were able to maintain an appropriate movement trajectory [6]. However, the unstable neuromuscular control pattern after stroke may compromise the ability, resulting in diminished motor performance. Indeed, the reduced wavelet coherence values in the patients, as opposed to controls, served as explicit evidence of the compromised muscle coordination mechanism.

Additionally, it is worth noting that the tracking errors of antagonist muscles mapped to the *x*-axis or *y*-axis in elbow flexion and extension did not reach statistical significance with the increase in dimensionality of MCI (1D→2D) in both controls and stroke patients. Statistically, the relatively small sample size limited the ability to detect differences in the visual dimensionality. Nevertheless, a consistent tendency of reduced tracking errors of the antagonist muscles was observed during 2D MCI in both controls and patients. This result aligned with previous findings [39,46,47] that subjects exhibited improved performance when given 2D instead of 1D visual feedback. A possible speculation about the reduced tracking errors was that 2D MCI promoted the motor correction and enhanced muscle coordination, thereby optimizing the tracking accuracy, through supplying more sufficient visual feedback information about antagonist muscles’ activation compared to 1D MCI.

On the muscle activation level, previous electrophysiology studies have found abnormal muscle activation patterns [13,48] and weak functional coupling [49,50,51] in stroke patients. Consistent with these findings, our results showed that stroke patients exhibited lower alpha- and beta-band wavelet coherence than the controls in the extension task. Owing to the fact that intermuscular coupling was a measure of the amount of common central drive to the muscles [52], the muscle-specific abnormality in functional coupling could be a reflection of inability to compensate for the reduced corticospinal input by compensatory mechanisms after stroke [53], which was reflected by the lower intermuscular coupling strength or the altered muscle networks [54,55]. Hence, the post-stroke impairment in motor control function weakened the intermuscular coupling of biceps and triceps. Regarding the alpha- and beta-band coherence, most studies on corticomuscular coherence have shown that low-frequency oscillations of the neuromuscular signal originated from the central nervous system [56,57]. Indeed, the EMG oscillations at the alpha band were derived from the motor cortex [19]. Another beta-band coherence originated from central motor neural pathways, reflecting the common central drive through an intact corticospinal tract [21,58,59]. However, in the lesioned hemisphere after stroke, the structural integrity of corticospinal tracts had diminished [60], and the motor area exhibited permanent and irreversible damage. Consequently, stroke patients exhibited weak intermuscular coherence compared to the controls, suggesting an impediment in the transmission of motor control information from the central nervous system (CNS). This may result in a reduction in cortical descending drive, thereby weakening muscle coupling.

### 4.2. Effect of MCI Dimensionality to the Intermuscular Coupling in Alpha and Beta Band

In the present study, wavelet coherence was utilized to analyze the dynamic intermuscular coupling across time and frequency domains. The integration in wavelet coherence reduced the correlation bias, enhancing the capacity of wavelet coherence to yield a more accurate localized correlation within the time–frequency domain [61]. Unlike conventional coherence method, wavelet coherence could effectively capture transient phenomena, thereby providing a comprehensive description of the EMG signals interactions.

The results showed that as MCI dimensionality increased, a significant decrease in the alpha-band coherence was only observed in the controls during the elbow extension. Nevertheless, a consistent decrease, though lacking statistical significance, was found in the stroke patients in the elbow extension task. The results collectively suggest that all participants could recognize the abnormal muscle coactivation and successfully decouple the target muscle pairs within 2D MCI. Consistent with our study, Watanabe et al. noted that increased visual feedback information was associated with decreased EMG–EMG coherence across all subjects [62]. Additionally, 2D MCI played an important role in diminishing the abnormal muscle coactivation in stroke patients [37,63]. These findings could be linked to a reduction in muscle coupling in response to increased visual feedback. Within 2D MCI, not only agonist activation but also antagonist activation was provided in real time during the task. The extra feedback of antagonist may help patients modulate the muscles’ activation and finally coordinate the muscle coupling better, which was observed as the decreased intermuscular coherence in 2D MCI.

As previously shown [19], the EMG oscillations at a range of 6 to 12 Hz originated from the motor cortex, suggesting a transmission of the alpha-band oscillations from the motor cortex to the muscles [64]. The brain activity at alpha band is considered to be related to visual attention and cognitive processing [65,66]. Increased visual feedback may cause the higher cognitive load [67,68]. Furthermore, a previous study showed that increased visual feedback may contribute to the motor error correction, reflected by an increased activation in most regions of the motor area [69]. The increased cortical activation in the primary motor cortex, in turn, could result in the decreased antagonist activation [37], indirectly influencing the intermuscular coupling. In the present study, a reduction in the alpha-band wavelet coherence was observed only in the controls with the increase in dimensionality in MCI. It is likely that increased visual feedback in 2D MCI during a tracking task facilitated the motor correction processing or cognitive processing, thus resulting in the influenced activity of the brain in the alpha band and the eventual affected alpha-band coherence. However, a similar reduction in the beta-band coherence was not significant in this study. In a previous study, the beta-band coherence was thought to be a pure cortical drive for the stable motor output, without relationship of cognition [70]. Consequently, our findings suggested that the cognition ability was an important factor in modulating the intermuscular coupling in the various conditions of different dimensionalities.

### 4.3. Clinical Implication and Limitations

Wavelet coherence exhibited superiority in discerning transient interactions between signals compared to traditional coherence, capable of indicating muscle coupling in both time and frequency domains during dynamic processes. In this study, the application of wavelet coherence revealed the effect of MCI dimensionality on the post-stroke intermuscular coupling during a tracking task. Interestingly, with additional visual feedback of the antagonist activation in 2D MCI, the controls and stroke patients adjusted muscle coupling patterns, possibly through descending neural pathways. These findings suggest the potential of wavelet coherence as a reliable biomarker for assessing the intermuscular coupling and further speculating on the corticomuscular interaction in the clinic. Furthermore, the rehabilitation training based on the MCI system may hold promise for reducing post-stroke motor abnormality and facilitating the recovery of muscle function. Future work would target a broader participant recruitment, encompassing individuals with mild, moderate, or severe stroke, to enhance comprehension regarding the potential contributions of the MCI system to the restoration of independent muscle function post-stroke.

## 5. Conclusions

In the present study, wavelet coherence was used to examine the intermuscular coupling between agonist and antagonist muscles in the controls and stroke patients during a dynamic tracking task. The 2D MCI could assist patients to adjust the muscle coupling by concurrently activating the agonist muscle and inhibiting the antagonist muscle. This finding implies that MCI holds promise as a novel tool for improving intermuscular coupling in stroke rehabilitation.

## Figures and Tables

**Table 1:** Detailed information of stroke patients.

Patients	Sex	Age	Lesion Side	Type of Stroke	Months after Stroke	UE-FMA Score (0–66)
Patient 1	M	53	L	Hemo	1.5	23
Patient 2	F	61	R	Hemo	18	47
Patient 3	F	53	L	Hemo	1.5	35
Patient 4	M	27	R	Isch	1	23
Patient 5	M	25	L	Isch	8	19
Patient 6	M	38	R	Isch	3	61
Patient 7	M	71	R	Hemo	12	54
Patient 8	M	52	L	Isch	5	51

Abbreviations: F, female; M, male; R, right; L, left; Isch, ischemic stroke; Hemo, hemorrhagic stroke; FMA-UE, Fugl–Meyer Assessment for Upper Extremity.

## Data Availability

The data presented in this study are available on request from the corresponding author due to ethical reasons.

## References

[1] Scott S.H. (2004). Optimal feedback control and the neural basis of volitional motor control. Nat. Rev. Neurosci..

[2] Zhu L.L., Lindenberg R., Alexander M.P., Schlaug G. (2010). Lesion load of the corticospinal tract predicts motor impairment in chronic stroke. Stroke.

[3] Kirker S.G.B., Simpson D.S., Jenner J.R., Wing A.M. (2000). Stepping before standing: Hip muscle function in stepping and standing balance after stroke. J. Neurol. Neurosurg. Psychiatry.

[4] Hidler J.M., Carroll M., Federovich E.H. (2007). Strength and coordination in the paretic leg of individuals following acute stroke. IEEE Trans. Neural Syst. Rehabil. Eng..

[5] Levin M.F., Solomon J.M., Shah A., Blanchette A.K., Feldman A.G. (2018). Activation of elbow extensors during passive stretch of flexors in patients with post-stroke spasticity. Clin. Neurophysiol..

[6] Kisiel-Sajewicz K., Fang Y., Hrovat K., Yue G.H., Siemionow V., Sun C.K., Jaskólska A., Jaskólski A., Sahgal V., Daly J.J. (2011). Weakening of synergist muscle coupling during reaching movement in stroke patients. Neurorehabilit. Neural Repair.

[7] Tian N., Chen Y., Sun W., Liu H., Wang X., Yan T., Song R. (2021). Investigating the stroke- and aging-related changes in global and instantaneous intermuscular coupling using cross-fuzzy entropy. IEEE Trans. Neural Syst. Rehabil. Eng..

[8] Yu H.R., Xu W.L., Zhuang Y., Tong K.Y., Song R. (2021). Wavelet coherence analysis of muscle coupling during reaching movement in stroke. Comput. Biol. Med..

[9] Wang L.-J., Yu X.-M., Shao Q.-N., Wang C., Yang H., Huang S.-J., Niu W.-X. (2020). Muscle fatigue enhance beta band EMG-EMG coupling of antagonistic muscles in patients with post-stroke spasticity. Front. Bioeng. Biotechnol..

[10] Luo J., Sun W., Wu Y., Liu H., Wang X., Yan T., Song R. (2018). Characterization of the coordination of agonist and antagonist muscles among stroke patients, healthy late middle-aged and young controls using a myoelectric-controlled interface. J. Neural Eng..

[11] Colamarino E., de Seta V., Masciullo M., Cincotti F., Mattia D., Pichiorri F., Toppi J. (2021). Corticomuscular and intermuscular coupling in simple hand movements to enable a hybrid brain–computer interface. Int. J. Neural Syst..

[12] Park J.H., Lee H., Kwon H.J., Shin J.H., Roh J., Park H.S. Feasibility of isokinetic training to modify coupling of upper limb muscle synergy activation in stroke-affected upper limb. Proceedings of the 2023 45th Annual International Conference of the IEEE Engineering in Medicine & Biology Society (EMBC).

[13] Dewald J.P.A., Pope P.S., Given J.D., Buchanan T.S., Rymer W.Z. (1995). Abnormal muscle coactivation patterns during isometric torque generation at the elbow and shoulder in hemiparetic subjects. Brain.

[14] Hammond M.C., Fitts S.S., Kraft G.H., Nutter P.B., Mj T., Lm R. (1988). Co-contraction in the hemiparetic forearm: Quantitative EMG evaluation. Arch. Phys. Med. Rehabil..

[15] Liu J.B., Wang J.X., Tan G.S., Sheng Y.X., Chang H., Xie Q., Liu H.H. (2021). Correlation evaluation of functional corticomuscular coupling with abnormal muscle synergy after stroke. IEEE Trans. Biomed. Eng..

[16] Farmer S.F., Gibbs J., Halliday D.M., Harrison L.M., James L.M., Mayston M.J., Stephens J.A. (2007). Changes in EMG coherence between long and short thumb abductor muscles during human development. J. Physiol..

[17] Santoso S., Powers E.J., Bengtson R.D., Ouroua A. (1997). Time-series analysis of nonstationary plasma fluctuations using wavelet transforms. Rev. Sci. Instrum..

[18] Qassim Y.T., Cutmore T.R.H., James D.A., Rowlands D.D. (2013). Wavelet coherence of EEG signals for a visual oddball task. Comput. Biol. Med..

[19] Marsden J.F., Brown P., Salenius S. (2001). Involvement of the sensorimotor cortex in physiological force and action tremor. Neuroreport.

[20] Wang L., Qiao M., Tao H., Song X., Shao Q., Wang C., Yang H., Niu W., Chen Y. (2022). A comparison of muscle activation and concomitant intermuscular coupling of antagonist muscles among bench presses with different instability degrees in untrained men. Front. Physiol..

[21] Farmer S.F., Bremner F.D., Halliday D.M., Rosenberg J.R., Stephens J.A. (1993). The frequency content of common synaptic inputs to motoneurones studied during voluntary isometric contraction in man. J. Physiol..

[22] Vallbo A.B., Wessberg J. (1993). Organization of motor output in slow finger movements in man. J. Physiol..

[23] Kilner J.M., Baker S.N., Salenius S., Jousmäki V., Hari R., Lemon R.N. (1999). Task-dependent modulation of 15-30 Hz coherence between rectified emgs from human hand and forearm muscles. J. Physiol..

[24] Grosse P., Cassidy M.J., Brown P. (2002). EEG–EMG, meg–EMG and EMG–EMG frequency analysis: Physiological principles and clinical applications. Clin. Neurophysiol..

[25] Delcamp C., Cormier C., Chalard A., Amarantini D., Gasq D. (2022). Changes in intermuscular connectivity during active elbow extension reveal a functional simplification of motor control after stroke. Front. Neurosci..

[26] Takahashi C.D., Der-Yeghiaian L., Le V., Motiwala R.R., Cramer S.C. (2007). Robot-based hand motor therapy after stroke. Brain.

[27] Malik A.N., Tariq H., Afridi A., Rathore F.A. (2022). Technological advancements in stroke rehabilitation. J. Pak. Med. Assoc..

[28] Cancel N., Fischer M., Gulotta L., Koo J., McKittrick S. (2014). Moderate aerobic exercise has an inconclusive effect on fine motor control. J. Adv. Stud. Sci..

[29] Charissou C., Vigouroux L., Berton E., Amarantini D. (2016). Fatigue- and training-related changes in ‘beta’ intermuscular interactions between agonist muscles. J. Electromyogr. Kinesiol..

[30] Charissou C., Amarantini D., Baurès R., Berton E., Vigouroux L. (2017). Effects of hand configuration on muscle force coordination, co-contraction and concomitant intermuscular coupling during maximal isometric flexion of the fingers. Eur. J. Appl. Physiol..

[31] Patel P., Kaingade S.R., Wilcox A., Lodha N. (2020). Force control predicts fine motor dexterity in high-functioning stroke survivors. Neurosci. Lett..

[32] Chen Y.-C., Lin L.L., Lin Y.-T., Hu C.-L., Hwang I.-S. (2017). Variations in static force control and motor unit behavior with error amplification feedback in the elderly. Front. Hum. Neurosci..

[33] Nguyen H.B., Lee S.W., Harris-Love M.L., Lum P.S. (2017). Neural coupling between homologous muscles during bimanual tasks: Effects of visual and somatosensory feedback. J. Neurophysiol..

[34] Farina D., Jiang N., Rehbaum H., Holobar A., Graimann B., Dietl H., Aszmann O.C. (2014). The extraction of neural information from the surface EMG for the control of upper-limb prostheses: Emerging avenues and challenges. IEEE Trans. Neural Syst. Rehabil. Eng..

[35] Mugler E.M., Tomic G., Singh A., Hameed S., Lindberg E.W., Gaide J., Alqadi M., Robinson E., Dalzotto K., Limoli C. (2019). Myoelectric computer interface training for reducing co-activation and enhancing arm movement in chronic stroke survivors: A randomized trial. Neurorehabilit. Neural Repair.

[36] Seo G., Kishta A., Mugler E., Slutzky M.W., Roh J. (2022). Myoelectric interface training enables targeted reduction in abnormal muscle co-activation. J. Neuroeng. Rehabil..

[37] Jian C.Y., Deng L.C., Liu H.H., Yan T.B., Wang X.Y., Song R. (2021). Modulating and restoring inter-muscular coordination in stroke patients using two-dimensional myoelectric computer interface: A cross-sectional and longitudinal study. J. Neural Eng..

[38] Jian C., Liu H., Deng L., Wang X., Yan T., Song R. (2021). Stroke-induced alteration in multi-layer information transmission of cortico-motor system during elbow isometric contraction modulated by myoelectric-controlled interfaces. J. Neural Eng..

[39] Kennedy M.W., Crowell C.R., Striegel A.D., Villano M., Schmiedeler J.P. (2013). Relative efficacy of various strategies for visual feedback in standing balance activities. Exp. Brain Res..

[40] Mista C.A., Christensen S.W., Graven-Nielsen T. (2015). Modulation of motor variability related to experimental muscle pain during elbow-flexion contractions. Hum. Mov. Sci..

[41] Missenard O., Mottet D., Perrey S. (2008). The role of cocontraction in the impairment of movement accuracy with fatigue. Exp. Brain Res..

[42] Gordon K.E., Ferris D.P. (2004). Proportional myoelectric control of a virtual object to investigate human efferent control. Exp. Brain Res..

[43] Rhif M., Ben Abbes A., Farah I.R., Martínez B., Sang Y. (2019). Wavelet transform application for/in non-stationary time-series analysis: A review. Appl. Sci..

[44] Espenhahn S., Rossiter H.E., van Wijk B.C.M., Redman N., Rondina J.M., Diedrichsen J., Ward N.S. (2020). Sensorimotor cortex beta oscillations reflect motor skill learning ability after stroke. Brain Commun..

[45] Zackowski K.M., Dromerick A.W., Sahrmann S.A., Thach W.T., Bastian A.J. (2004). How do strength, sensation, spasticity and joint individuation relate to the reaching deficits of people with chronic hemiparesis?. Brain.

[46] Mordkoff J.T., Danek R.H. (2011). Dividing attention between color and shape revisited: Redundant targets coactivate only when parts of the same perceptual object. Atten. Percept. Psychophys..

[47] Young S.J., van Doornik J., Sanger T.D. (2011). Visual feedback reduces co-contraction in children with dystonia. J. Child Neurol..

[48] Fellows S.J., Kaus C., Thilmann A.F. (1994). Voluntary movement at the elbow in spastic hemiparesis. Ann. Neurol..

[49] Larsen L.H., Zibrandtsen I.C., Wienecke T., Kjaer T.W., Christensen M.S., Nielsen J.B., Langberg H. (2017). Corticomuscular coherence in the acute and subacute phase after stroke. Clin. Neurophysiol..

[50] Houston M., Li X.Y., Zhou P., Li S., Roh J., Zhang Y.C. (2021). Alterations in muscle networks in the upper extremity of chronic stroke survivors. IEEE Trans. Neural Syst. Rehabil. Eng..

[51] Canning C.G., Ada L., O’Dwyer N.J. (2000). Abnormal muscle activation characteristics associated with loss of dexterity after stroke. J. Neurol. Sci..

[52] Delcamp C., Gasq D., Cormier C., Amarantini D. (2023). Corticomuscular and intermuscular coherence are correlated after stroke: A simplified motor control?. Brain Commun..

[53] Rossini P.M., Calautti C., Pauri F., Baron J.C. (2003). Post-stroke plastic reorganisation in the adult brain. Lancet Neurol..

[54] Du Y., Yang W., Yao W., Qi W., Chen X., Xie B., Xie P. (2019). Analysis of multichannel intermuscular coupling characteristics during rehabilitation after stroke. J. Biomed. Eng..

[55] Houston M., Li R., Roh J., Zhang Y. Altered muscle networks in post-stroke survivors. Proceedings of the 2020 42nd Annual International Conference of the IEEE Engineering in Medicine & Biology Society (EMBC).

[56] Volkmann J., Joliot M., Mogilner A.Y., Ioannides A.A., Lado F.A., Fazzini E., Ribary U., Llinás R.R. (1996). Central motor loop oscillations in parkinsonian resting tremor revealed magnetoencephalography. Neurology.

[57] Mima T., Hallett M. (1999). Corticomuscular coherence: A review. J. Clin. Neurophysiol..

[58] Power H.A., Norton J.A., Porter C.L., Doyle Z., Hui I., Chan K.M. (2006). Transcranial direct current stimulation of the primary motor cortex affects cortical drive to human musculature as assessed by intermuscular coherence. J. Physiol..

[59] Fisher K.M., Zaaimi B., Williams T.L., Baker S.N., Baker M.R. (2012). Beta-band intermuscular coherence: A novel biomarker of upper motor neuron dysfunction in motor neuron disease. Brain.

[60] Karbasforoushan H., Cohen-Adad J., Dewald J.P.A. (2019). Brainstem and spinal cord mri identifies altered sensorimotor pathways post-stroke. Nat. Commun..

[61] Li X., Yao X., Fox J., Jefferys J.G. (2007). Interaction dynamics of neuronal oscillations analysed using wavelet transforms. J. Neurosci. Methods.

[62] Watanabe T., Nojima I., Mima T., Sugiura H., Kirimoto H. (2020). Magnification of visual feedback modulates corticomuscular and intermuscular coherences differently in young and elderly adults. NeuroImage.

[63] Wright Z.A., Rymer W.Z., Slutzky M.W. (2014). Reducing abnormal muscle coactivation after stroke using a myoelectric-computer interface: A pilot study. Neurorehabilit. Neural Repair.

[64] Salmelin R., Hari R. (1994). Characterization of spontaneous meg rhythms in healthy adults. Electroencephalogr. Clin. Neurophysiol..

[65] Cooper N.R., Croft R.J., Dominey S.J.J., Burgess A.P., Gruzelier J.H. (2003). Paradox lost? Exploring the role of alpha oscillations during externally vs. Internally directed attention and the implications for idling and inhibition hypotheses. Int. J. Psychophysiol..

[66] Kranczioch C., Debener S., Maye A., Engel A.K. (2007). Temporal dynamics of access to consciousness in the attentional blink. NeuroImage.

[67] Puh U., Vovk A., Sevsek F., Suput D. (2007). Increased cognitive load during simple and complex motor tasks in acute stage after stroke. Int. J. Psychophysiol..

[68] Meester D., Al-Yahya E., Dawes H., Martin-Fagg P., Piñon C. (2014). Associations between prefrontal cortex activation and h-reflex modulation during dual task gait. Front. Hum. Neurosci..

[69] Archer D.B., Kang N., Misra G., Marble S., Patten C., Coombes S.A. (2018). Visual feedback alters force control and functional activity in the visuomotor network after stroke. NeuroImage Clin..

[70] Pogosyan A., Gaynor L.D., Eusebio A., Brown P. (2009). Boosting cortical activity at beta-band frequencies slows movement in humans. Curr. Biol..

